# New Targets of Neuroprotection in Ischemic Stroke

**DOI:** 10.1100/tsw.2008.94

**Published:** 2008-07-13

**Authors:** Marco Bacigaluppi, Dirk M. Hermann

**Affiliations:** ^1^ Department of Neurology, University Hospital Zurich (USZ), CH-8091 Zurich, Switzerland; ^2^ Neuroimmunology Unit and Institute of Experimental Neurology IRCCS San Raffaele Milano, I-20132 Milano, Italy; ^3^ Vascular Neurology, Dementia and Ageing Research, Department of Neurology, University Hospital Essen, D-45122 Essen, Germany

**Keywords:** stroke, pathophysiology, animal models, reperfusion, neuroprotection, neurorestoration

## Abstract

Ischemic stroke represents one of the most challenging diseases in translational neurology. Despite the considerable efforts made to develop efficacious therapies that prevent damage once a stroke has occurred, there are still no established treatments for humans. The only available treatment is intravenous or intra-arterial thrombolysis that is limited to the very first hours after the stroke. Starting with recent findings about the pathophysiology of stroke and presenting an overview on current experimental models of this clinically highly relevant neurological disease, this paper will provide an overview on existing and emerging treatment concepts in ischemic stroke. Thus, our review will present an analysis of established and innovative strategies of neuroprotection and neurorestoration, highlighting both pharmacological and cell-based treatment concepts. In the last section of our paper, we will examine more closely how experimental data are presently translated to humans, with particular emphasis on the bioaccumulation and efficacy of drugs. From this point of view, we will try to develop ideas of how causative treatments in ischemic stroke may be established.

## INTRODUCTION

Ischemic stroke represents the third-frequent cause of mortality and leading cause of long-term disability in industrialized countries. The number of people affected annually by stroke, actually over 2 million worldwide, is estimated to increase dramatically in the next years and to increase by almost one-half by 2020. This is because of the increase in the mean population age, the persistence of unhealthy habits, and the emerging risk factors that will affect young patients in particular[1,2] (World Health Organization, http://www.who.int/cardiovascular_diseases/resources/atlas/en). These epidemiological aspects have prompted research to study and develop animal models to recreate the complex pathophysiology of ischemic stroke to possibly find efficacious therapeutic approaches.

Despite the important insights that have been made in understanding the complex pathophysiology of stroke in the last 25 years, today the only efficacious treatment approved for ischemic stroke is thrombolysis. Unfortunately only 5–10% of ischemic stroke patients can be elected to this treatment because of its limited time window and its potentially dangerous side effects[1,3]. Since the 1980s, research strategies have focused on two main approaches other than recanalization of occluded vessels, which is often not feasible: neuroprotection, to reduce cell death and infarct volume after stroke, and neural repair, to facilitate the reorganization and the reconstruction of damaged neural tissue. While the therapies developed in experimental research showed good efficacy in animal models, none of these were proved to date to have efficacy in clinical trials[4].

This review will comprehensively summarize basic aspects of ischemic stroke pathophysiology, critically evaluate current experimental models that are used in basic research, and provide an overview on established and emerging neuroprotective and neurorestoration therapies. Current concepts of preclinical and clinical study design (as presented by the Stroke Academic Industry Roundtable [STAIR] guidelines), together with our own ideas and reflections regarding future research strategies, which should establish smoother transitions from animal to human studies[5,6], will also be discussed.

## PATHOPHYSIOLOGY OF STROKE

A critical reduction of cerebral blood flow to a localized part of brain tissue that persists over time and causes neuropathological changes is defined as ischemic stroke. Different causes can underlie a stroke, the most frequent being atherosclerosis of small or large vessels, cardiogenic embolisms, vasculopathies associated with dissection of brain-supplying arteries, vasculitis, and disorders of the coagulation system[7]. Whatever the different causes that trigger the occlusion of a brain vessel, the inadequate blood supply initiates a series of pathophysiological events that, if not reversed over time, result in irreversible damage of brain tissue. The critical reduction can be a consequence of the inadequate blood flow distal to occluded arteries, which mostly indicates insufficient collateralization by surrounding vessels. As such, blood flow is too low to maintain metabolic processes required for preserving structural and functional brain integrity[8].

In this context, the threshold concept of brain hemodynamics deserves attention. Cerebral blood flow exhibits dynamic flow thresholds in the ischemic brain evolving over space and time. At moderate reductions of blood flow, the functional activity of the brain is first impaired ("electrical failure") and the synthesis of new proteins is suppressed. In principle, this reversible impairment is followed, at more severe degrees of blood supply deprivation, by the failure of cellular energy state and the loss of electrical membrane potentials ("membrane failure")[9]. After a very short delay, the energy failure converts into irreversible structural brain injury.

The area of brain tissue exhibiting energy failure is usually referred to as infarct core, while the tissue revealing functional failure is called penumbra. The distinction of these zones, penumbra and core, is of great importance, as the penumbra is a potential salvable brain region that over time evolves into core, i.e., dead tissue, if blood flow is not re-established in a timely manner[10,11,12]. The zone of penumbra is the conceptual basis for therapeutic interventions in the acute stroke phase either with thrombolytic or neuroprotective drugs. Both interventions are, in fact, focused on rescuing the penumbral zone from transformation into core[13].

To understand the pathophysiology of stroke and therapeutic opportunities, it is reasonable to subdivide the evolution of stroke into three main phases. The first phase is characterized by the energy failure and resulting depolarization of cell membranes that takes place in the center of an infarct within seconds to minutes when blood flow is compromised. The second phase, in which the infarct grows, lasts up to 6–72 h after occlusion[10,11], depending on the severity and duration of ischemia. Its main feature is the expansion of the infarct core into the penumbra. The underlying mechanisms that lead to infarct expansion are spreading depressions, excitotoxicity, calcium release, formation of free radicals, endoplasmic reticulum dysfunction, and mitochondrial failure. The third phase is the chronic phase that lasts for several days and also weeks after injury. It is characterized by a plethora of secondary injury mechanisms, including cytotoxic and vasogenic edema, inflammation, and delayed cell death that further contribute to the progression of tissue damage[9]. Importantly, this phase is also characterized by endogenous repair mechanisms that are aimed at restoring and clearing the suffered damage and that can be resumed by the attempt of the tissue to regain lost functions through plasticity processes (intended are all processes that lead to a functional reorganization, either neurogenesis, synaptogenesis, or angiogenesis)[14].

The injured brain releases a variety of regeneration-enabling molecules in the surrounding area of the brain damage. Unfortunately, these growth-promoting factors are counteracted in the adult brain by inhibitory influences preventing regeneration. First of all, adult neurons have intrinsic limitations, compared to embryonic neurons, to translate regeneration signals into successful sprouting[15]. However, even more important than the aborted regeneration temptations are extrinsic growth inhibitory cues that can be divided in three main classes: CNS myelin-specific inhibitory factors; glial scar components released by reactive astrocytes, including chondroitin sulfate proteoglycans; and cytokines that exert a double-edged effect (both promoting and inhibiting) on CNS regeneration[15,16]. In the section "Neurorestorative Treatments", we will discuss in more detail some of the many pharmacological approaches that have been tried in experimental research in order to overcome the inhibitory cues.

A detailed discussion of the mechanisms that synergistically contribute to the final tissue damage following stroke is out of the scope of the present review (excellent reviews can be consulted: see [8,9]). The adequate understanding of the pathophysiology of stroke is important in order to conceive specific therapeutic approaches and to define appropriate time windows for them. The administration of a specific therapy at the wrong time point can, in fact, have injurious effects. These effects, although not always dramatic as in the case of delayed thrombolysis that leads to massive injuries, can nonetheless impair the physiological repair mechanisms of the brain. As such, the inhibition of the metalloproteinase-9 (MMP-9), which is efficacious in the very acute phase after ischemic stroke[17,18], was shown to block neurovascular remodeling when performed in the chronic phase (i.e., 7–14 days after stroke)[19]. On the other hand, the too early administration of regeneration-promoting therapies may also be potentially detrimental for tissue survival, as exemplified by our own still-unpublished experiments in which the deactivation of the neurite growth inhibitor NogoA with monoclonal antibodies was detrimental for tissue survival, when delivered before the stroke. This underlines once more the concept that the success of specific treatments in stroke is time dependent[20].

## EXPERIMENTAL STROKE MODELS

In the 1980s, relevant animal models of cerebral ischemia were developed in order to identify the mechanisms that cause tissue damage and to develop new therapies for stroke at a preclinical level. Today, reliable animal models for ischemic stroke are available in a number of different species, including primates, dogs, cats, pigs, Mongolian gerbils, rabbits, rats, and mice (Table 1). Small animals are actually the preferred species to perform preclinical studies because they are suited for ischemic strokes, physiological variables can be easily monitored, and sufficient numbers for statistical analyses can be reached without excessive costs[21]. The different types of methods developed to create strokes in the rodents reflect the complexity of the disease and the need of different paradigms to optimize the study of different therapeutic approaches.

Stroke models can be divided into two main categories: models of global and of focal cerebral ischemia (Table 1). The first is induced by occluding the four main supplying vessels of the brain (the two carotid and vertebral arteries) or inducing cardiac arrest, while the latter can further be created in different ways: by proximal occlusion of the middle cerebral artery (pMCAo), by distal occlusion of the MCA (dMCAo), by photothrombosis using injection of Rose Bengal dye followed by illumination to produce a cortical lesion, or by direct injection of embolic material (sphere or thrombin clots) into one of the main cerebral vessels (Table 1). Models of global cerebral ischemia are used to study brain damage that occurs in cardiocirculatory resuscitation and mimics mechanisms that are not always relevant in focal strokes.

**TABLE 1**
**Animal Models of Experimental Global and Focal Cerebral Ischemia**

| Animal Species | Type of Ischemia | Stroke Induction Method | Ref. |
|---|---|---|---|
| Nonhuman primates (squirrel, macaque, and baboon monkey) | Global | Four-vessel occlusion by neck cuff inflation | [87] |
| | | Innominate and left subclavian arteries occlusion | [88] |
| | Focal | Occlusion M1 segment of the MCA with:<br>(1) intracranial approach along the sphenoidal wing;<br>(2) retro-orbital approach;<br>(3) transorbital approach | <br>[89]<br>[90]<br>[91] |
| | | Anterior cerebral artery occlusion | [92] |
| Dog | Global | Cardiac arrest/resuscitation model | [93] |
| | | Four-vessel occlusion by vessel clamping or neck cuff inflation | [94] |
| | Focal | Cerebral embolism by microfibrillar collagen injection | [95] |
| | | MCAo | [96] |
| | | Brainstem infarction | [97] |
| Cat | Global | Four-vessel occlusion by neck cuff inflation plus hypotension technique | [98] |
| | | Innominate and left subclavian arteries occlusion | [99] |
| | Focal | MCAo by retro-orbital approach | [100] |
| | | Snare ligature around the MCA | [101] |
| Pig | Global | Cardiac arrest/resuscitation model | [102] |
| | Focal | Transorbital distal MCAo | [103] |
| Gerbil | Global | Bilateral carotid artery occlusion | [104] |
| | | Unilateral carotid artery occlusion | [105] |
| Rabbit | Focal | Rabbit small clot embolus model | [106] |
| Rodent (rat, mouse) | Global | Neck tourniquet | [107] |
| | | Cardiac arrest/resuscitation | [108] |
| | | Bilateral common carotid artery occlusion (CCAo) combined with controlled ventilation | [109] |
| | | Four-vessel occlusion model | [110] |
| | | Two-vessel occlusion model | [111] |
| | Focal | Proximal MCAo in the rat and in the mouse | [112,113,114] |
| | | Distal MCAo in the rat and in the mouse | [25,26] |
| | | Three-vessel occlusion model | [115] |
| | | MCAo + ipsilateral common carotid artery occlusion | [116] |
| | | Photothrombosis model | [27,117] |
| | | Thromboembolic macrosphere injection model | [28] |
| | | Thromboembolic microsphere injection model | [29] |
| | | Thromboembolic clot injection model | [118] |

pMCAo models belong to the most frequently used procedures in stroke research. pMCAo is usually induced by advancing a suture into the internal carotid artery to occlude the MCA at its origin from the circle of Willis. The severity of ischemia can be modulated simply by leaving the suture filament in place either permanently or transiently for a variable duration of time (often between 30–120 min), after which the suture is then withdrawn, which results in tissue reperfusion. This procedure has the advantage

(whether the same occlusion time is performed) that it is highly reproducible. It does not require craniotomy (avoiding its complications) and produces focal damages as in embolic human stroke[12,22]. Short-lasting pMCAo causes selective neuronal death in the lesion-sided striatum, expression of heat shock proteins, immediate early gene expression, and induction of apoptotic signal pathways[23,24]. Longer durations of occlusion instead result in brain infarcts that involve both the striatum and overlying cortex[10], and may be associated with some animal mortality in case the tissue swells. The latter complication is frequently observed in animals, in which brain injury develops in deep brain structures comprising the thalamus and midbrain, which is inevitable at least in some of the animals. The latter pattern of injury clearly reflects a disadvantage of intraluminal MCA occlusion models.

dMCAo is usually performed via a craniotomy to occlude superficial branches of the MCA directly. The damage is, compared to the pMCAo model, more restricted, spares deep brain structures as the striatum and thalamus, but can sometimes be complicated by side effects due to the craniotomy (subarachnoidal bleeding, cerebral infection, mechanical induction of spreading depression, or cerebrospinal fluid leakage)[25,26]. Photothrombosis is induced by the transcranial illumination of the brain after systemic delivery of a photosensitive dye (Rose Bengal) that results in the coagulation of the irradiated tissue[27]. This model has the advantage that the region of ischemia can be predefined, opening the possibility to coagulate distinct cortical areas with great precision. The disadvantages of this model are the nonphysiological insult (photocoagulation) that creates a small lesion without clearly defined penumbra.

As human stroke is most frequently caused by cerebral thromboembolism, a number of animal models have been developed that closely mimic the embolic occlusion of brain vessels. Embolic strokes can be induced in animals through injection of large-sized macrospheres (300–400 μm in diameter) or small-sized microspheres (<50 μm) into the internal carotid artery. In the first case, large infarcts similar to those produced by the permanent occlusion of the MCA are induced. In the latter case, smaller, multifocal infarcts are observed[28,29].

To study thrombolytic therapies (a clinically very relevant therapeutic issue), a third model of vascular occlusion has been developed using autologous blood clots that are injected directly into the internal carotid artery[30,31,32,33,34]. While embolic stroke models produce rather variable infarcts that make it more difficult to test neuroprotective therapies, they are well suited to study reperfusion therapies[35]. In view of their variability, thromboembolic stroke models have achieved limited significance in neuroprotection research in the past, which may represent a reason for the translation failure of animal data to humans. In fact, mechanical occlusion models poorly reflect the hemodynamic aspects of thrombolytic reperfusion, which potentially might lead to an altered responsiveness of the brain tissue to neuroprotective treatments. The latter idea is supported by observations regarding the progression of metabolic disturbances and energy failure after cerebral thromboembolism followed by thrombolysis in mice[36], which differed from intraluminal MCA occlusion findings[10,11,24].

In view of many negative clinical neuroprotection trials, guidelines regarding preclinical and clinical stroke research were published in 1999 by an academic roundtable bringing together stroke specialists as well as researchers from the pharmaceutical industry, aiming to improve research strategies and increase the efficacy of stroke studies[2]. These recommendations questioned the validity of hitherto used research strategies, stimulating scientists to test new drugs in a more comprehensive way in different animal models and across species barriers. The STAIR guidelines also postulated that more precise evaluations of dose-response relationships should be made and time windows of drug efficacy should be determined. They also encouraged the inclusion of functional readouts into animal studies in addition to morphological data analysis. Despite the valuable influence of the STAIR guidelines on the standards of stroke studies performed in the meantime, the translation of animal findings to humans has still remained unsuccessful in recent years. The precise reasons for the study failure remain a mystery. It is obvious, however, that the interface between animal and human studies still has to be optimized[5]. As such, it is a major problem that we mostly conduct animal research in young laboratory animals, whereas the vast majority of stroke patients are elderly and sometimes exhibit severe atherosclerotic disease. In addition, the design of human trials exhibits a number of serious pitfalls, relating to time windows, the selection of endpoints (morphological vs. functional neurological), as well as drug dosage [5]; see also below).

## TREATMENT CONCEPTS IN ISCHEMIC STROKE

The efficacy of stroke therapies strongly depends on the time point at which a treatment is applied. As such, treatment success is strictly time dependent. Three overall approaches can be distinguished, their utility depending on the phase at which the therapy is initiated.

### Reperfusion Therapies

The first approach aims at reperfusion. It attempts to reinstall blood flow before irreversible tissue damage develops. Reperfusion of an occluded vessel can be achieved by delivering a thrombolytic agent (either via the intravenous or intra-arterial route) that dissolves the blood clot, or by mechanically trapping or dissolving the occluding embolus, which is often done in conjunction with arterial thrombolysis. Reperfusion therapy nowadays represents the cornerstone of modern stroke therapy, and it is a sine-qua-non requirement for tissue survival, as without reperfusion the tissue is destined to die. Major limitations of thrombolysis are the limited time window in which reperfusion therapies can be initiated (up to 3 to maximally 4.5 h after stroke in humans based on a recent meta-analysis; see [37,38]) and the presence of exclusion criteria (e.g., medically uncontrollable hypertension, anticoagulation, recent surgical interventions, presence of signs of hemorrhage in computerized tomography scans), which rule out that thrombolysis can be offered to stroke patients in a broader way. As such, no more than 5–10% of patients may receive this treatment even in specialized stroke units[1,39,40]. Recently, potentially detrimental effects of thrombolytic drugs, such as tissue-plasminogen activator (t-PA) in the reperfused brain parenchyma have been a concern. In the extracellular space of the brain parenchyma, t-PA indeed contributes to matrix degradation following stroke (reviewed by Hermann and Matter[41]), resulting in a secondary derangement of tissue integrity affecting both brain hemodynamics[35] and neuronal survival[42]. Recently, it has been shown that add-on treatments are able to prevent t-PA's injurious effects[43]. In this context, cholesterol-lowering statins have achieved particular attention[44,45]. Whether this so-called t-PA toxicity is also relevant in humans and whether more selective plasminogen activators that are presently evaluated in clinics may prevent it, still needs to be awaited.

### Neuroprotective Therapies

The second therapy approach to acute stroke is neuroprotection. It attempts to block key aspects of the ischemic cascade in order to prevent ischemic brain tissue from evolving into infarcted tissue[46,47,48]. The delivery and efficacy of neuroprotective agents relies on the existence of ischemic brain tissue that is still not irreversibly injured. In principle, this time window extends up to approximately 24–72 h after stroke onset, depending on whether ischemia is permanent or reversible. Yet, it needs to be realized that the amount of viable tissue rapidly decreases within the first few hours after stroke. As such, within 6 h after permanent ischemia, large parts of the penumbra are already lost[11]. Therefore, treatments should be initiated as soon as possible (golden rule: "time is brain"). As neuroprotective agents do not confer a similarly high adverse risk as thrombolytics (particularly regarding brain bleedings), neuroprotectants can be applied over a longer time span (up to days after stroke).

The cascade of injury in case of ischemic stroke is, as discussed above, very complex and multifaceted. Different neuroprotective agents have been developed to block the progression of the injury signal at different steps. However, the neuroprotective effects depend on various influence factors, such as the time point after the onset of stroke, the persistence of blood flow interruption, the presence or absence of secondary injury mechanisms (such as spreading depressions or free radical stress), as well as the temperature of the brain[4,49]. In the last 20 years, over 1000 compounds have been tested in animal models of ischemic stroke, but of the 114 compounds that have successively entered clinical trials, none has been successful in clinical practice until now[50,51].

In view of the failure of neuroprotection trials, recent studies have focused on naturally occurring molecules, such as the sexual hormone estrogen, the anti-inflammatory compound minocycline, the endogenous growth factors erythropoietin (Epo), vascular endothelial growth factor (VEGF) or granulocyte colony-stimulating factors (G-CSF), or the free radical scavenger melatonin. Moreover, physical-chemical approaches such as hypothermia or hyperbaric oxygen delivery attempt to increase neuronal survival, rebalancing the disturbed neuronal energy budget (Table 2). Compared with compounds that act on single cellular pathways, growth factors have the clear advantage of their pleiotropic action, which targets several cell signaling pathways inside injured cells. Besides, these compounds not only have survival-promoting actions on neurons, but also on glial and vascular cells.

**TABLE 2**
**Endogenous Neuroprotective Strategies Recently Used for Ischemic Stroke Treatment**

| Neuroprotective Agent | Mechanism of Neuroprotection | Ref. |
|---|---|---|
| Estrogens and analogues | Inhibition of apoptosis, modulation of calcium homeostasis, direct antioxidant activity | [119,120,121,122] |
| Minocycline | Anti-inflammatory effect, reduction of microglial activation, reduction of MMP activation, reduction of nitric oxide, inhibition of apoptotic cell death | [123,124,125,126,127,128] |
| Epo | Inhibition of apoptosis, anti-inflammatory effect | [129,130,131,132,133] |
| VEGF | Inhibition of apoptosis, reduction of nitric oxide, induction of angiogenesis and neurogenesis | [134,135,136,137] |
| G-CSF | Anti-inflammatory effect, inhibition of apoptosis, induction of angiogenesis, increased neuronal differentiation | [138,139,140] |
| Melatonin | Inhibition of apoptosis, reduction of nitric oxide, anti-inflammatory effect, induction of neurogenesis | [55,56,57,141] |
| Hypothermia | Prevention of blood brain barrier disruption, decreased oxygen free radical production, lowering of metabolic rate and energy depletion, anti-inflammatory effect | [142,143,144] |
| Hyperbaric oxygen | Increased $O_2$ delivery to the tissue, augmentation of cerebral blood flow | [145,146] |

Interestingly, growth factors not only promote cell survival, but also stimulate the remodeling of the brain tissue by enhancing angiogenesis and supporting axonal regeneration. Interactions between neurons and vascular cells have received particular interest in the recent past, as it was realized that both cell types exhibit mutual, trophic interactions that promote restorative processes in the stroke brain (the term "neurovascular niche" was coined for this mutual relationship)[52,53]. Unfortunately, most of the growth factors have a big molecular weight and due to their hydrophilicity are largely unable to pass the blood brain barrier (erythropoietin, Epo: 30,000 Da; nerve growth factor, NGF: 30,000 Da; fibroblast-growth factor-2, FGF-2: 24,000 Da; ciliary neurotrophic factor, CNTF: 24,000 Da; epidermal growth factor, EGF: 6,045 Da). The development of small-molecule mimics of growth factors may open new perspectives so that these strategies may be used clinically. Compared with growth factors, melatonin is, for its high lipophilicity and its low molecular size (molecular weight 232.2 Da), an interesting candidate for stroke treatment, as it readily passes the blood brain barrier on administration[54]. Melatonin was indeed shown to have good pharmacokinetic characteristics and to have both direct neuroprotective effects (it inhibits apoptosis by reducing nitric oxide, by the anti- inflammatory effect) and neuroregenerative effects, as it induces neurogenesis[54,55,56,57].

## Neurorestorative Treatments

The third strategy, neurorestoration, approaches the latest injury phase, characterized by the activation of inflammatory responses, scar formation, and formation of new neuronal circuits (either micro- and/or macroscopic) that allow compensation of lost functions. The time window for this treatment is by far longer than that for recanalizing and neuroprotective strategies. It expands over many days to weeks after stroke, thus giving the opportunity to treat a larger number of stroke patients. The rationale of this treatment is to augment and sustain the intrinsic brain repair properties and to enhance the structural and functional reorganization of the brain subjected to stroke[58]. Although a full regeneration after ischemic injury of the brain would require the replacement of lost neurons and the formation of neuronal projections over long distances, as well as the regeneration of the lost glial, vascular, and extracellular matrix structure, lost functions often can partly be compensated by axonal sprouting from spared neurons and by rearrangement of surviving circuits[15].

The peri-infarct milieu is characterized by various different major regenerative events such as axonal sprouting aimed at forming new connections and the establishment of novel projection patterns that are hampered by inhibitory molecules[59]. The cortical reorganization that occurs after CNS damage and that leads to some spontaneous recovery[60] can be manipulated therapeutically. Particularly promising molecules in this context are myelin-associated neurite growth inhibitors, among which the 35 and 250 kDa proteins named Nogo recently received great attention. Nogo is a member of the reticulon family of membrane proteins, and three different isoforms (Nogo-A, -B, and -C) are generated by alternative splicing and promoter usage[61]. Of the three isoforms, Nogo-A is the best characterized, as it is highly expressed by CNS oligodendrocytes[62,63,64].

After extensive research *in vitro* showing that inhibition of Nogo-A by means of monoclonal antibodies (e.g., the monoclonal antibody IN-1 and the Nogo-A antibody 7B12) could partially neutralize the growth inhibitory effect of CNS myelin and oligodendrocytes leading to enhanced neurite outgrowth, *in vivo* experiments were performed[65,66]. The application of the very same antibodies in diverse models of diseases of the CNS as stroke and spinal cord injury demonstrated that, also *in vivo*, these antibodies antagonize Nogo-A's antiregenerative action, as enhanced neurite outgrowth on neuropathological studies and also functional recovery of animals with spinal cord lesions or ischemic stroke in rodents[66,67,68] was observed. In view of encouraging data regarding both structural regenerative responses in lesioned corticospinal pathways, as well as functional improvements in neurological motor tests, clinical trials with Nogo-A antibodies are presently on their way. Apart from these highly promising data with Nogo-A antibodies, growth factors have been used to promote the remodeling of the brain tissue. In this context, VEGF, basic fibroblast growth factor (bFGF), brain-derived neurotrophic factor (BDNF), G-CSF, Epo, and phosphodiesterase type 5 (PDE-5) inhibitors have been studied[58].

Besides growth factors, cell-based therapies have recently been used as therapeutics promoting brain repair in animal models of stroke injury[69,70]. Namely, bone marrow–derived stem cells and neural precursor cells were used. The therapeutic potential of immature cells is mediated less by their capacity to differentiate into various differentiated cell lineages (including astrocytes, neurons, oligodendrocytes, and endothelial cells), resulting in replacement of lost brain tissue, than by their ability to secrete anti-inflammatory cytokines and trophic factors into their environment that directly support angiogenesis, neurogenesis, and synaptic plasticity in the infarct border zone[58]. Although some clinical trials were made using cellular therapies in stroke patients in the 1980s and 1990s — these studies used cell lines that were still poorly characterized and lacked adequate controls[69] — further experimental studies dissecting the precise actions of, as well as clarifying, safety concerns are needed before new clinical trials should be made. Safety studies should particularly rule out that cell therapies do not result in an elevated tumor risk.

## DELIVERY OF THERAPEUTICS TO THE STROKE BRAIN

Besides the mechanisms of action of therapeutics used for ischemic stroke treatment, the delivery of pharmacological compounds, growth factors, antibodies, and cell-based therapies deserves attention. Pharmacological compounds are mostly administered systemically via the intravenous or subcutaneous route. Thus, all compounds delivered systemically need to pass the blood brain barrier in order to reach their target tissue. The blood brain barrier is formed by capillary endothelial cells, which are interconnected by tight junctions[71,72,73]. Tight junctions strongly restrict paracellular permeability of water-soluble molecules between the vascular lumen and brain parenchyma. This prevents the entrance of hydrophilic molecules to the brain tissue, unless they are carried transcellularly, either via transcytosis or receptor-dependent uptake[73]. Indeed, the ability of pharmacological compounds to pass the blood brain barrier most strongly depends on a molecule's lipophilicity. As such, lipophilic compounds most readily accumulate inside the brain[6].

Unfortunately, lipophilic compounds do not pass into the brain without encountering a shuttle mechanism, formed by transmembranous ATP-binding cassette (ABC) transporters, which actively eliminate the drugs from the brain[6,74,75]. ABC transporters bind a large number of chemically unrelated pharmacological molecules[6,76], suggesting that their biological role is to keep the brain clean from hazardous environmental influences. Among known ABC transporters, the transporter ABCB1 (previously called multidrug resistance transporter-1) has the broadest substrate affinity, extruding up to 50% of all pharmacological compounds from the brain[77]. Since ABCB1 is selectively up-regulated in ischemic brain tissue[78], the transporter impedes the accumulation of drugs in the ischemic brain tissue. In view of its eminent role in drug biodistribution, the pharmaceutical industry has developed selective inhibitors of ABCB1[6], which have recently become available in clinics. ABCB1 inhibitors particularly increase the accumulation of neuroprotective drugs in the ischemic brain[78], raising hopes that overall drug efficacy may be enhanced. The biodistribution of drugs was an under-recognized issue in translational stroke research in the past. Accumulation aspects should receive increased attention, particularly at the interface between animal and human studies. In this context, it is important to notice that expression patterns of drug transporters exhibit considerable differences between species[79]. This may have a profound influence on the accumulation of drugs in the brain[6].

Compared with small-molecule pharmacological drugs, growth factors, antibodies, or even cells have much greater difficulty entering the brain tissue. In case of antibody-based therapies, intrathecal delivery strategies are, therefore, often advantageous[80]. However, even after intrathecal delivery, antibodies mostly accumulate in areas close to the cerebrospinal fluid compartment, which in bigger mammals, such as primates and humans, implies that the antibody mostly accumulates in superficial brain areas. It is surprising, therefore, that the plasticity-promoting Nogo-A antibodies enhanced functional neurological recovery in monkeys subjected to spinal cord trauma[81]. The beneficial effects of Nogo-A antibodies may be explained by their growth-promoting effect on superficial corticospinal tract fibers. Whether this strategy similarly leads to functional improvements after hemispheric stroke in humans, in which the pyramidal tract lesion is located deep inside the brain, still needs to be checked.

In contrast to peptides and antibodies, therapeutic cells have the ability to migrate actively into the brain tissue, which after stroke is facilitated by overexpression of chemoattractant cues, attracting and guiding the transplanted cells towards tissue injury[82,83,84]. Studies in experimental autoimmune encephalitis have shown that the chemoattractants CCL5/RANTES and CXCL12/SDF-1 play a key role in the recruitment of adult neural precursor (so-called stem) cells to the injured brain[85]. Interestingly, adult neural precursor cells mostly remain in an undifferentiated state in the brain tissue. This largely precludes that these cells act via cell replacement. It is more likely that these cells release growth factors and anti-inflammatory mediators, which in turn promote the remodeling of the brain tissue and enhance structural neuroplasticity of uninjured brain areas leading to functional recovery[86].

## CONCLUSIONS

In view of its complex pathophysiology, which affects blood vessels, neurons, and glial cells, the quick translation of experimental discoveries into clinical practice has previously raised problems. Yet, this clearly does not imply that the development of treatments is without prospect. Research in the past years has demonstrated that research efforts should not only focus on single-cell pathways, but also target multiple signal cascades and make use of the endogenous restorative potential of the brain, which may be activated with trophic factors, antibodies, or cell-based therapies. As such, research findings have pointed out that different types of brain cells (neurons, glia, vascular cells) exhibit mutual interactions with each other, which may be used in order to promote survival and facilitate tissue remodeling. Restorative treatments have recently allowed us to extend time windows considerably, and besides pharmacological agents, antibody and cell-based therapies have been established. Although the last decade was still characterized by the failure of several clinical trials with innovative pharmacological drugs, the advancements in our understanding of basic stroke pathophysiology, the improvements in the design of basic and clinical research studies, as well as insights into pharmacological principles represent achievements that should make it possible that causative therapeutic strategies may soon find their way into clinics.

## ACKNOWLEDGMENTS

Ertugrul Kilic and Luca Peruzzotti
